# Loss of Function of the Nuclear Receptor *NR2F2*, Encoding COUP-TF2, Causes Testis Development and Cardiac Defects in 46,XX Children

**DOI:** 10.1016/j.ajhg.2018.01.021

**Published:** 2018-02-22

**Authors:** Anu Bashamboo, Caroline Eozenou, Anne Jorgensen, Joelle Bignon-Topalovic, Jean-Pierre Siffroi, Capucine Hyon, Attila Tar, Péter Nagy, Janos Sólyom, Zita Halász, Annnabel Paye-Jaouen, Sophie Lambert, David Rodriguez-Buritica, Rita Bertalan, Laetitia Martinerie, Ewa Rajpert-De Meyts, John C. Achermann, Ken McElreavey

**Affiliations:** 1 Human Developmental Genetics, Institut Pasteur, Paris 75724, France; 2 Department of Growth and Reproduction, Rigshospitalet, Copenhagen 2100, Denmark; 3 Department of Medical Genetics, Hospital Trousseau-APHP, Paris 75012, France; 4 Heim Pál Children’s Hospital, Budapest 1089, Hungary; 5 First Department of Pathology and Experimental Cancer Research, Semmelweis University, Budapest, Hungary; 6 Department of Pediatrics, Semmelweis University, Budapest 1085, Hungary; 7 Pediatric and Visceral Surgery and Urology, Hopital Robert Debre, Paris 75019, France; 8 Endocrinologie et Diabetologie Pediatrique, Hopital Robert Debre, Paris 75019, France; 9 Division of Genetics, Department of Pediatrics, McGovern Medical School, University of Texas, Houston, TX 77030, USA; 10 UCL GOSH Institute of Child Health, London WC1N 1EH, UK

**Keywords:** sex determination, COUP-TF2, NR2F2, nuclear receptor, testicular DSD, new syndrome

## Abstract

Emerging evidence from murine studies suggests that mammalian sex determination is the outcome of an imbalance between mutually antagonistic male and female regulatory networks that canalize development down one pathway while actively repressing the other. However, in contrast to testis formation, the gene regulatory pathways governing mammalian ovary development have remained elusive. We performed exome or Sanger sequencing on 79 46,XX *SRY*-negative individuals with either unexplained virilization or with testicular/ovotesticular disorders/differences of sex development (TDSD/OTDSD). We identified heterozygous frameshift mutations in *NR2F2*, encoding COUP-TF2, in three children. One carried a c.103_109delGGCGCCC (p.Gly35Argfs^∗^75) mutation, while two others carried a c.97_103delCCGCCCG (p.Pro33Alafs^∗^77) mutation. In two of three children the mutation was *de novo*. All three children presented with congenital heart disease (CHD), one child with congenital diaphragmatic hernia (CDH), and two children with blepharophimosis-ptosis-epicanthus inversus syndrome (BPES). The three children had androgen production, virilization of external genitalia, and biochemical or histological evidence of testicular tissue. We demonstrate a highly significant association between the *NR2F2* loss-of-function mutations and this syndromic form of DSD (p = 2.44 × 10^−8^). We show that COUP-TF2 is highly abundant in a FOXL2-negative stromal cell population of the fetal human ovary. In contrast to the mouse, these data establish COUP-TF2 as a human “pro-ovary” and “anti-testis” sex-determining factor in female gonads. Furthermore, the data presented here provide additional evidence of the emerging importance of nuclear receptors in establishing human ovarian identity and indicate that nuclear receptors may have divergent functions in mouse and human biology.

## Main Text

The sex chromosomes of an individual usually direct gonadal sex development toward either a testis or ovary pathway. In 46,XY individuals, the presence of the Y chromosome testis-determining gene *SRY* (MIM: 601947) triggers a genetic cascade that both initiates testis formation and represses the formation of the ovary.^1^ Although many factors are known to be involved in early testis development, far less is known about genetic factors controlling the ovary.^1^ However, emerging evidence suggests that ovary development involves more than just a *default* (passive) pathway,^2^ consistent with the *Z-factor theory* proposed 25 years ago that the XX gonad expresses an elusive factor that actively promotes both “anti-testis” and “pro-ovary” functions.^3^

The genomic analysis of 46,XX individuals with testes (sometimes known as testicular disorders/differences of sex development [TDSD]; [MIM: 400045]) or ovotestes (ovotesticular DSD [OTDSD]) supports the hypothesis that “pro-testis/anti-ovary” or “pro-ovary/anti-testis” genetic pathways exist.^4^, ^5^, ^6^ These children typically present with virilized genitalia due to testosterone production from the presence of testicular tissue. Many individuals with TDSD and a minority with OTDSD have a translocation of the testis-determining *SRY* gene, usually onto one of the X chromosomes, whereas a small proportion have chromosomal rearrangements associated with upregulation (gain-of-function) of *SOX* gene expression (e.g., duplications of the *RevSex* [MIM: 616425] enhancer of *SOX9* [MIM: 608160] and rearrangements of the *SOX3* locus [MIM: 800833]).^5^, ^6^ This ectopic *SOX* gene expression increases “pro-testis” factors in the early developing ovary and results in a reprogramming of cellular sex identity to testis-typical Sertoli cells. Other rare forms of 46,XX DSD can occur due to mutations (loss-of-function) involving genes that are “pro-ovary/anti-testis.” The best example of this is in the WNT4/RSPO1 signaling pathway, which stabilizes β-catenin and is required for ovary development.^7^ Very rare heterozygous or homozygous mutations in *WNT4* (MIM: 603490) cause Müllerian aplasia/hyperandrogenism (MIM: 158330)^8^ or testis development (SERKAL syndrome [MIM: 611812]) in 46,XX individuals, respectively, whereas homozygous *RSPO1* (MIM: 609595) mutations are also associated with testis formation and skin phenotypes in 46,XX children (MIM: 610644).^7^ FOXL2 (MIM: 605597) is a forkhead/winged helix transcription factor, which is also a potential “pro-ovary” and “anti-testis” gene. Goat XX fetuses lacking *Foxl2* have gonads resembling testes and the induced absence of *Foxl2* in adult XX mice results in *trans*-differentiation of the ovaries into a testis-like gonad.^9^, ^10^, ^11^ In humans, heterozygous loss-of-function *FOXL2* mutations are associated with autosomal-dominant blepharophimosis-ptosis-epicanthus inversus syndrome (BPES [MIM: 110100]) either with (type I) or without (type 2) ovarian insufficiency as well as ovarian insufficiency without BPES (MIM: 608996), but homozygous *FOXL2* disruption has not been described.^10^ The limited understanding of the genes involved in ovary formation means that for most children with OTDSD/TDSD, the molecular basis is currently unknown.

Here, we report the molecular etiology of a syndromic form of 46,XX DSD that includes genital virilization, congenital heart disease (CHD), and variable somatic anomalies including BPES and congenital diaphragmatic hernia (CDH). This syndrome is caused by protein-truncating mutations in the orphan nuclear receptor *NR2F2* (MIM: 107773). *NR2F2* encodes the transcription factor chicken ovalbumin upstream promoter transcription factor 2 (COUP-TF2).^12^ Our data indicate that in the early developing human chromosomal female XX gonad, COUP-TF2 is a human “pro-ovary” and “anti-testis” sex-determining factor.

Following written informed consent and with local ethics committee approvals, whole-exome sequencing was performed on 69 46,XX individuals with either unexplained virilization (n = 14) or unexplained OTDSD/TDSD (n = 55) as described elsewhere.^13^ Sanger sequencing of *NR2F2* was performed on DNA from a further 10 individuals with 46,XX OTDSD/TDSD where there was insufficient DNA for complete exome analysis. Exclusion criteria included rearrangements involving *SOX* genes, mutations in either *WNT4* or *RSPO1*, proven or suspected congenital adrenal hyperplasia (CAH), or the presence of *SRY*. All samples underwent array comparative genomic hybridization to confirm normal ploidy. Of the 79 children studied, 74 had no additional syndromic features. Of the 5 children with additional features, 3 were unrelated children with OTDSD/TDSD and CHD (with or without BPES), one had 46,XX TDSD with cerebral leukodystrophy, and another had 46,XX TDSD with multiple congenital anomalies, learning difficulties, and alopecia.

**Figure 1:**
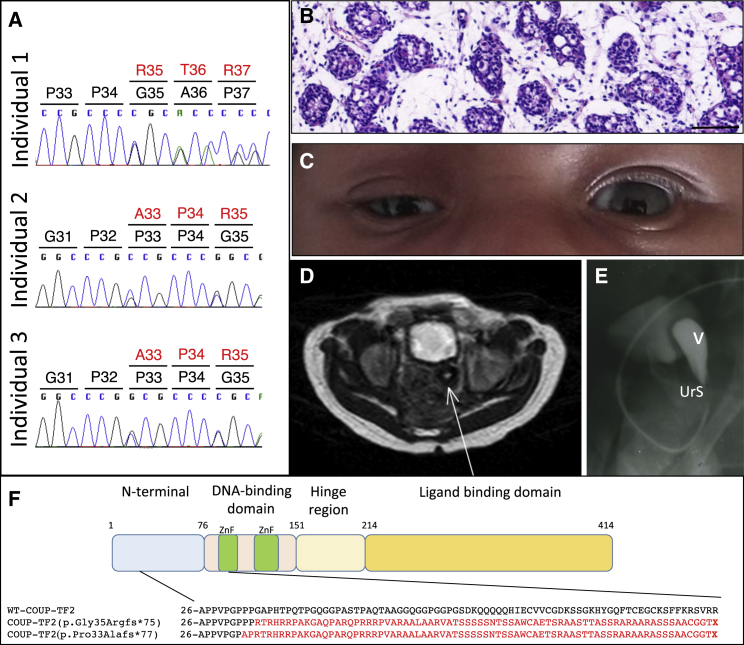
Identification of Three Individuals with 46,XX DSD and Heterozygous Frameshift Mutations in *NR2F2* (A) Representative Sanger sequence chromatograms of individuals 1–3 showing the positions of the frameshift mutations. (B) Histology of the right gonad of individual 3 showing testicular tubule-like structures surrounded by stromal-like tissue (scale bar corresponds to 100 μm). (C) Blepharophimosis-ptosis-epicanthus inversus syndrome (BPES) of individual 3. (D) Uterus (arrow) of individual 2 observed by MRI. (E) Pelvic radiography of individual 3 showing the vagina (V) and a short urogenital sinus (UrS). (F) Schematic representation of COUP-TF2 showing the main functional domains and the position and downstream consequences of the three frameshift mutations. The first zinc finger motif is highlighted in green in the sequence alignment. The transcript ID is GenBank: NM_021005.3.

Analysis of exome sequencing datasets identified two individuals (individuals 1 and 2) with near-identical heterozygous frameshift mutations in *NR2F2* (GenBank: NM_021005.3), c.103_109delGGCGCCC (p.Gly35Argfs^∗^75), and c.97_103delCCGCCCG (p.Pro33Alafs^∗^77) (Table 1). These mutations were not present in parental DNA and hence are *de novo*. No other shared genes carrying *de novo* mutations were identified in the datasets. The mutations are predicted to result in the generation of a downstream termination codon (Figures 1A and 1F). Both children presented with suspected OTDSD/TDSD (Table 1) and congenital heart disease (CHD). Individual 1 had a left congenital diaphragmatic hernia (CDH) and died shortly after birth due to hypoplasia of the left heart. Analysis of an additional 10 individuals with 46,XX OTDSD/TDSD, for whom there was insufficient DNA for complete exome studies, revealed a third unrelated child with OTDSD and CHD harboring the c.97_103delCCGCCCG (p.Pro33Alafs^∗^77) mutation (Table 1, individual 3). Since the parents of this child were unavailable for study, the mode of transmission of the mutation is unknown. The diagnosis of 46,XX OTDSD was confirmed by histology of the gonad (Figure 1B). Individuals 2 and 3 also had BPES (Figure 1C). In all three children, potentially pathogenic mutations were not identified in any other genes known to be involved in DSD and high-resolution array comparative genomic hybridization (aCGH) did not indicate rearrangements involving DSD-associated genes. These frameshift mutations are absent from the public variant databases and they were detected only in the three individuals in the cohort who had cardiac anomalies together with 46,XX DSD.

**Table 1:** Phenotypes, Genotypes, and Investigation of Three Children with Frameshift Mutations in *NR2F2*

**Variable**	**Individual 1**	**Individual 2**	**Individual 3**
Ancestry	Latino	Senegalese	Hungarian
Karyotype	46,XX *SRY*-negative	46,XX *SRY*-negative	46,XX *SRY*-negative
Birth weight (kg)	2.6	2.05	N/A
Cardiac	hypoplastic L heart at birth; severely dilated R ventricle with moderate RVH and mild to moderately depressed RV systolic function	persistent ostium secundum and ASD	VSD at birth; spontaneous closure of VSD at 9 years of age
External genitalia	male, hyperpigmented; phallus 3 cm with no hypospadias; gonads not palpable	“ambiguous” with phallus-like clitoris, pigmented scrotum; gonads not palpable	“ambiguous” Prader IV, pigmented scrotum, phallus-like clitoris; R, palpable gonad in the inguinal canal
Internal genitalia	uterus not identified by US	uterus present	R, ductus-like Wolffian structures; L, uterus, ovary, and Fallopian tube; vagina and short urogenital sinus
Gonads	not observed by US	not observed by US or MRI	pelvic ultrasound: R testis, L ovary; histology: R gonad, testis tubules, and ovarian tissue with oocytes
Other somatic anomalies	left congenital diaphragmatic hernia	BPES	mild learning disabilities, minor limb anomalies, hypertelorism, BPES
Endocrine data (reference values)	Day 1: T, 135 ng/dL (20–64); 17-OHP, 120 ng/dL (11–170); LH, 5.1 IU/L (0.02–7.0); FSH, 1.1 IU/L (0.16–4.1)	Day 11: T, 579 ng/dL (<130); AMH, 44 ng/mL (<4.2); Inhibin B, 263 pg/mL (<110); FSH, 13.2 IU/L (<10); LH, 9.3 IU/L (0.9–3); 17-OHP, 127 ng/dL (<270) Day 15: T, 327 ng/dL (<40); AMH, 43.1 ng/mL (<4.2); Inhibin B, 217 pg/mL (<110); FSH, 6.9 IU/L (<10); LH, 5.1 IU/L (0.9–3); estradiol <5 pg/mL 1 month: T, 304 ng/dL (<40); AMH, 43.1 ng/mL (<4.2); Inhibin B, 230 pg/mL (<110); FSH, 9 IU/L (<10); LH, 15.5 IU/L (0.9–3); estradiol, <5 pg/mL	Day 17: T, 250 ng/dL (<40); 17-OHP, 185 ng/dL (40–490) Day 30: T, 460 ng/dL (<40) Day 45: T, 50 ng/dL (<40) Day 73: T, 110 ng/dL (<40)
Mutation	*de novo*, c.103_109delGGCGCCC (p.Gly35Argfs^∗^75)	*de novo*, c.97_103delCCGCCCG (p.Pro33Alafs^∗^77)	N/A, c.97_103delCCGCCCG (p.Pro33Alafs^∗^77)

Normal range refers to the range of basal levels in control subjects matched according to age and chromosomal sex with the case subjects. Conversion factors: testosterone ng/dL x 0.034 for nmol/L; 17-OHP ng/dL x 0.0303 for nmol/L; AMH ng/mL x 7.14 for pmol/L; estradiol pg/mL x 3.67 for pmol/L.

Abbreviations: L, left; R, right; RVH, right ventricular hypertrophy; US, ultrasound; T, testosterone; 17-OHP, 17-hydroxyprogesterone; LH, luteinizing hormone; FSH, follicle-stimulating hormone; ASD, atrial septal defect; MRI, magnetic resonance imaging; BPES, blepharophimosis ptosis epicanthus inversus syndrome; AMH, anti-Müllerian hormone; VSD, ventricular septal defect; N/A, not available. Individual 1 had a male-typical phenotype and high testosterone consistent with testicular tissue; individual 2 had high AMH and inhibin B and high testosterone consistent with testicular tissue; individual 3 had high testosterone and histological evidence of testicular tissue. In all situations, biochemical and genetic testing for other causes of virilization such as congenital adrenal hyperplasia (e.g., 17-OHP) were negative.

*NR2F2* is one the most conserved genes in the human genome and is intolerant to protein-truncating variants.^14^ To exclude the possibility that these *NR2F2* mutations may be a chance finding, we performed Fisher’s exact tests (two-tailed) on the frequency of protein-truncating mutations found in the case subjects and compared this to rare missense mutations from 6,489 control European American and African American cohorts in the NHLBI-ESP project. A rare variant is defined as a variant with an allelic frequency of <1% in publically available databases. We found no protein-truncating mutations present in the control populations. We found a highly significant enrichment for the *NR2F2* protein-truncating mutations identified in the three 46,XX DSD-affected case subjects compared to the absence of protein-truncating mutations in the control cohort (Fisher’s exact test, two-tailed p = 1.1 × 10^−6^). Even if we consider the five missense mutations present in the control population, the finding of three frameshift mutations in the 46,XX DSD cohort is still highly significant (p = 6 × 10^−5^). The genetic evidence in favor of causality is more compelling when one considers that the protein-truncating mutations were specifically identified *only* in the three children presenting with CHD and 46,XX DSD (p = 2.44 × 10^−8^).

*NR2F2* encodes a key nuclear receptor, COUP-TF2.^12^ All three frameshift mutations are present in the N-terminal region of the protein and result in a downstream premature termination codon. RNA containing these mutations is predicted to be degraded by nonsense-mediated decay. Even if a truncated protein is produced, it would have severely impaired biological activity since the protein would lack both the DNA- and ligand-binding domains (Figure 1F). An *in silico* analysis of the *NR2F2* exon 1 with either of the two deletions using the tools Human Splicing Factor 3 (HSF3), Splice Predictor, and RegRNA indicated that neither the two deletions are predicted to generate cryptic splice sites.

**Figure 2:**
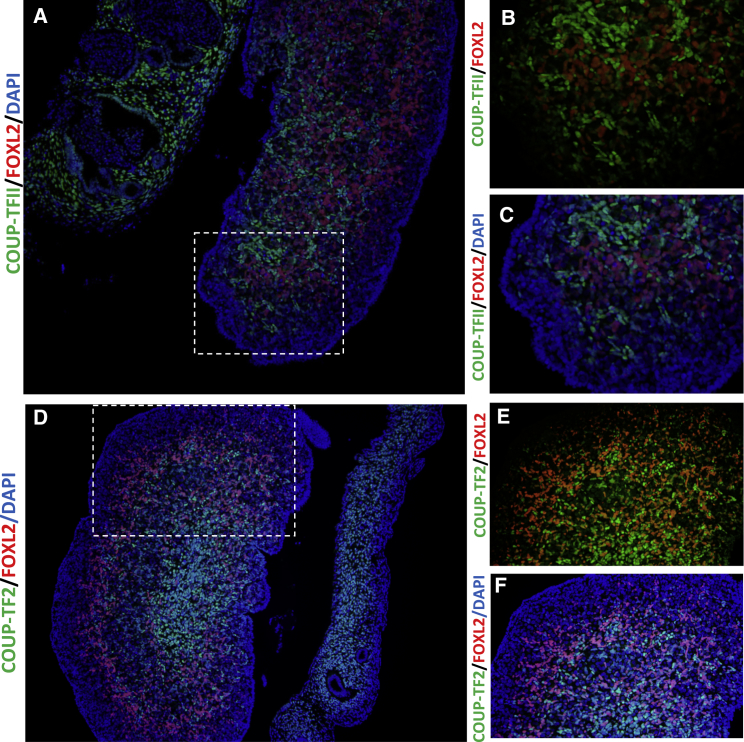
Protein Localization of COUP-TF2 and FOXL2 during Early Human Ovarian Development (A–C) Immunofluorescence showing protein localization of COUP-TF2 (green) and FOXL2 (red) at gestational week (GW) 9+1. (D–F) Immunohistochemistry at GW 9+5. Extensive staining of COUP-FT2 is observed in the stromal cell population of the developing fetal ovary. At both stages there appears to be a mutually exclusive presence of FOXL2 and NR2F2, suggesting they mark different somatic cell populations. Dashed box in (A) and (C) indicates the position of the expanded views. Nuclei are counterstained with DAPI. Fetal age was determined by scanning crown-rump length and by evaluation of foot length.^34^ Sex of fetal samples was determined by PCR for *SRY* as previously reported.^35^ Immunofluorescence was performed as previously described.^36^ Primary antibodies used were COUP-TFII/NR2F2 (Perseus Proteomics, PP-H7147-60, diluted 1:100) and FOXL2 (a kind gift from Dagmar Wilhelm, diluted 1:100). Negative controls were included and processed with the primary antibody replaced by the dilution buffer alone. None of the negative control slides showed staining. Fluorescent images were captured using an Olympus BX61 microscope (Olympus) with the Cell Sens Dimension software v.1.16.

Murine Coup-tf2 protein has been reported to accumulate during early ovary development in a distinct Foxl2-negative somatic cell precursor.^15^ Although data are limited, these cells with Coup-tf2 have been hypothesized to be either mesenchymal cells and/or to differentiate into theca cells.^15^ We therefore investigated the profile of protein localization of COUP-TF2 in the early developing human ovary. Human fetal ovaries were isolated from material available following elective termination of pregnancy during the first trimester at the Department of Gynaecology at Copenhagen University Hospital (Rigshospitalet) and Hvidovre Hospital, Denmark. The regional ethics committee approved this study (permit number H-1-2012-007) and women gave their informed written and oral consent. We observed widespread presence of COUP-TF2 in the stromal cell population of the ovary at gestational weeks (GW) 9+1 (Figures 2A–2C) and GW 9+5 (Figures 2D and 2E), whereas FOXL2 is limited to somatic cells of the fetal ovary (Figure 2). At these stages, co-immunofluorescence demonstrated that FOXL2 and COUP-TF2 appeared to be mutually exclusive at the cellular level (Figure 2). Furthermore, analysis of RNA expression in different tissues from adults reveals that *NR2F2* is most highly expressed in the ovary and female reproductive tissues.^16^

We provide compelling genetic evidence for a role of COUP-TF2 in early human ovary development. We identified three protein-truncating, complete loss-of-function mutations that are acting in a dominant fashion. These findings indicate that *NR2F2* may function in a dosage-sensitive manner, which is similar to other gene mutations known to disrupt human sex determination. This indicates that COUP-TF2 may be required to establish ovary identity during early human gonad development by the repression of genes involved in testis determination.

In mice, Coup-Tf2 is involved in the development of multiple organs and tissues by modulating the expression of downstream targets to promote cellular differentiation, proliferation, migration, survival, and intercellular communication.^12^, ^17^, ^18^, ^19^, ^20^ Globally, Coup-tf2 is highly abundant at E14-E15 in the mesenchymal compartment of the developing organs and declines after the completion of organogenesis.^12^ The absence of Coup-tf2 in terminally differentiated epithelium suggests that Coup-tf2 plays a major role in the mesenchymal-epithelial transition. Mice lacking Coup-tf2 die around E10 due to defects of angiogenesis and heart development.^20^ Recently, ten human heterozygous missense, in-frame duplication, or splice donor mutations in *NR2F2* were reported in association with cardiac defects (CHD [MIM: 615779]).^21^, ^22^ Of these mutations, four were confirmed as *de novo*. Most children with *NR2F2* mutations had atrioventricular septal defects, including a child with a hypoplastic left heart, which is consistent with the cardiac phenotypes of the children in this study. However, non-cardiac defects, such as virilization or BPES, were not described in these cohorts. In our study, individual 1 also presented with a left-sided diaphragmatic hernia (CDH). The p.Pro33Alafs^∗^77 variant was reported in a child with ASD, exotropia, and left-sided CDH, suggesting that CDH is part of the phenotypic spectrum associated with *NR2F2* mutations.^23^

The BPES phenotype observed in children with *NR2F2* mutations is strikingly similar to that associated with dominant mutations in *FOXL2*. These pathogenic variants cause BPES frequently together with 46,XX premature ovarian insufficiency but testis development has not been reported. BPES is thought to originate following developmental defects in peri-ocular mesenchymal cells, which are required to form the levator smooth muscle, tarsus, and Meibomian glands. Coup-tf2 is known to play crucial roles in the formation of the morphology of the murine eye, and given the similarities in the phenotypes, it would be of interest to determine whether FOXL2 and COUP-TF2 are acting in the same developmental pathway.^24^

In contrast to cardiac development, the role(s) of COUP-TF2 in early gonad development has yet to be fully defined. In the developing fetal rat testis, partial inhibition of Coup-tf2 by phthalates causes expansion of Leydig cells and an increase in the intratesticular testosterone concentration, consistent with Coup-tf2 being a factor repressing fetal androgenization.^19^ The protein localization of COUP-TF2 in human fetal Leydig cells during the first and second trimester could also be consistent with a similar role of COUP-TF2 in repression of androgen production.^19^, ^25^ Indeed, there is a large body of evidence from early studies to indicate that COUP-TF2 functions as a transcriptional repressor of pro-testis genes.^26^

Data are also emerging for the role of Coup-Tf2 in female reproductive development in mice but there seem to be clear differences with humans. Murine *Coup-Tf2*^+/−^ XX females show a wide range of reproductive anomalies including reduced fecundity, irregular estrus cycles, delayed puberty, retarded postnatal growth, and reduced levels of steroidogenic enzymes, but virilization and testis development has not been reported.^27^ Recently, a tamoxifen-inducible Wt1^*CreERT2*^ mouse model that targets *Nr2f2* in Wt1^+^ mesenchymal cells was described, where knockout XX mice had both Müllerian and Wolffian ducts in the mesonephros.^28^ The ovaries of the mice lacking *Nr2f2* did not produce androgens, but an androgen-independent activation of the p-ERK pathway in the Wolffian duct epithelium was observed, leading to its maintenance in XX mice. In striking contrast to the mouse model, virilization of the genitalia in the three children reported here was associated with elevated levels of both testosterone and AMH and in one child the presence of testicular tissue was confirmed by gonad histology.

The mechanism responsible for testis development associated with COUP-TF2 variants remains to be defined. Consistent with the *Z*-theory of a double repressor model of human sex determination, COUP-TF2 acts both positively and negatively to modulate the expression of genes involved in sex determination in other cellular and organ contexts. For example, in the human endometrium, COUP-TF2 is a positive regulator of the pro-ovary *WNT4* gene and in mice Coup-tf2 negatively regulates the expression of the pro-testis *Sox9* gene in the osteogenic mesenchyme.^29^, ^30^

In addition to revealing unforeseen mechanisms in sex development, our study also highlights the importance of nuclear receptors (NR) in human development and disease. Humans have 48 different NRs with a diverse range of functions and next-generation sequencing approaches are starting to identify novel and sometimes unexpected phenotypes associated with specific NR variants.^31^ Recently, we described recurrent missense mutations that specifically target the Arg92 residue of another nuclear receptor, NR5A1 and which are associated with both 46,XX OTDSD and TDSD in multiple individuals.^32^ When this mutation is introduced into the XX mouse, it does not result in testis formation.^33^ The data presented here provide additional evidence of the emerging importance of nuclear receptors in establishing human ovarian identity and indicate that nuclear receptors may have divergent functions in mouse and human biology.

## Web Resources

ExAC Browser, http://exac.broadinstitute.org/
GenBank, https://www.ncbi.nlm.nih.gov/genbank/
Human Splicing Finder, http://www.umd.be/HSF3/
NetGene2, http://www.cbs.dtu.dk/services/NetGene2/
NHLBI Exome Sequencing Project (ESP) Exome Variant Server, http://evs.gs.washington.edu/EVS/
OMIM, http://www.omim.org/
RegRNA, http://regrna.mbc.nctu.edu.tw/html/prediction.html
The Human Protein Atlas, https://www.proteinatlas.org/ENSG00000185551-NR2F2/tissue
